# Antagonism of Host Antiviral Responses by Kaposi's Sarcoma-Associated Herpesvirus Tegument Protein ORF45

**DOI:** 10.1371/journal.pone.0010573

**Published:** 2010-05-11

**Authors:** Fan Xiu Zhu, Narayanan Sathish, Yan Yuan

**Affiliations:** 1 Department of Biological Science, Florida State University, Tallahassee, Florida, United States of America; 2 Department of Microbiology, School of Dental Medicine, University of Pennsylvania, Philadelphia, Pennsylvania, United States of America; Yale University, United States of America

## Abstract

Virus infection of a cell generally evokes an immune response by the host to defeat the intruder in its effort. Many viruses have developed an array of strategies to evade or antagonize host antiviral responses. Kaposi's sarcoma-associated herpesvirus (KSHV) is demonstrated in this report to be able to prevent activation of host antiviral defense mechanisms upon infection. Cells infected with wild-type KSHV were permissive for superinfection with vesicular stomatitis virus (VSV), suggesting that KSHV virions fail to induce host antiviral responses. We previously showed that ORF45, a KSHV immediate-early protein as well as a tegument protein of virions, interacts with IRF-7 and inhibits virus-mediated type I interferon induction by blocking IRF-7 phosphorylation and nuclear translocation (Zhu et al., Proc. Natl. Acad. Sci. USA. 99:5573-5578, 2002). Here, using an ORF45-null recombinant virus, we demonstrate a profound role of ORF45 in inhibiting host antiviral responses. Infection of cells with an ORF45-null mutant recombinant KSHV (BAC-stop45) triggered an immune response that resisted VSV super-infection, concomitantly associated with appreciable increases in transcription of type I IFN and downstream anti-viral effector genes. Gain-of-function analysis showed that ectopic expression of ORF45 in human fibroblast cells by a lentivirus vector decreased the antiviral responses of the cells. shRNA-mediated silencing of IRF-7, that predominantly regulates both the early and late phase induction of type I IFNs, clearly indicated its critical contribution to the innate antiviral responses generated against incoming KSHV particles. Thus ORF45 through its targeting of the crucial IRF-7 regulated type I IFN antiviral responses significantly contributes to the KSHV survival immediately following a primary infection allowing for progression onto subsequent stages in its life-cycle.

## Introduction

Kaposi's sarcoma-associated herpesvirus (KSHV), also known as human herpesvirus 8 (HHV-8), is a human DNA tumor virus belonging to the gamma herpesviridae family [1], [2]. It represents one of the latest additions to the DNA tumor virus group and is etiologically associated with Kaposi's sarcoma (KS), a malignant neoplasm of endothelial origin [1]. KSHV is also unequivocally associated with certain lymphoproliferative disorders, namely primary effusion lymphoma (PEL) and multicentric Castleman's disease (MCD) [3], [4].

The infectious cycle of herpesviruses starts with the virus binding to the cell surface followed by the release of the viral components into the cell. The host cell responds to these events through the creation of an antiviral state, predominantly associated with the synthesis and secretion of soluble type I interferon (IFN-α/β), that make up vital components of the host innate immune system [5], [6]. This is exemplified in related herpesviruses, like herpes simplex virus (HSV) and human cytomegalovirus (HCMV), wherein the binding of the respective envelope glycoproteins, gD and gB, to the cell surface receptors, trigger the host cell antiviral responses through type I IFN signaling [7], [8]. In KSHV also, the initial interaction of the viral glycoprotein gpK8.1 with cell surface components was shown to trigger the type I IFN pathway [9].

In spite of the above observation, following primary infection of cultured cells with KSHV, there was no significant induction of type I IFNs or their receptors [10]. Further the antiviral response elicited by gpK8.1 was clearly inhibited by infection of cells with UV-treated KSHV virions [9]. These findings clearly indicated that a possible virion component efficiently disarms the host immune response immediately following viral infection. Such a phenomenon is also seen with related herpesviruses like HCMV and rhesus CMV (RhCMV) where the tegument components likely play pivotal roles in inhibition of the host immune responses [11]–[13]. In HSV-1 also a similar antagonism of the host innate immune responses is seen which however occurs only following viral gene expression [14]. Avoidance of the initial host antiviral state thus allows to virus to successfully establish an infection allowing its further progression down the viral life-cycle.

The type I IFNs modulating the innate antiviral response trigger the transcription of the IFN-stimulated genes (ISGs) which through multiple pathways target the invading virus thus significantly reducing the virus burden [15], [16]. Type I IFN transcription is primarily controlled by two IFN regulatory factors (IRFs), IRF-3 and IRF-7 [17]–[19]. The constitutively expressed IRF-3, upon initial viral infection, undergoes activation and induces expression of IFN-β (early phase). This initial induction of IFN-β stimulates the type I IFN receptor resulting in the activation of IRF-7, which efficiently induces both the IFN-α and the IFN -β genes initiating a positive feedback loop (late phase) [17]–[19]. Though this classical pathway reveals an important role of IRF-7 only in the later stages of type I IFN induction, recent studies using IRF-7 deficient (IRF-7^−/−^) mice have provided for a significantly different picture [20], [21]. Accordingly the endogenously expressed, low level IRF-7 (in uninfected cells) gets activated following a primary viral invasion and brings about the initial induction of type I IFN. This subsequently further activates IRF-7 initiating a positive feedback loop aimed at robust late stage induction of IFNs. Thus by participating in both the initial and the late phases of type I IFN secretion, IRF-7 emerged as the predominant factor controlling type I IFN mediated innate immune responses [20], [21].

A new and exciting pathway for the robust induction of type I IFNs also involves activation of the toll like receptor (TLR) molecules following viral invasion [20], [21]. This is typified by the activation of TLR9 sub-family of proteins that subsequently recruits an adaptor protein, the myeloid differentiation primary response gene 88 (MyD88). This adaptor protein through exclusive interaction with and activation of IRF-7, results in type I IFN secretion, governing the induction of CD8^+^ T-cell responses contributing to adaptive immunity in dendritic cells (DCs) [20], [21]. This observation gains more value in the light of certain studies suggesting that DCs could be infected with KSHV and could possibly play vital roles in the transmission and pathogenesis of KSHV [22], [23].

From the preceding observations it becomes quite evident that IRF-7 is the master regulator of the host immune responses, controlling both innate and adaptive immunity. Thus given the significance of IRF-7 in the induction of immune responses and the fact that a virion component of KSHV does disarm the host immune response, our attention turned to the study of the candidate virion component proteins that participate in the selective inhibition of IRF-7 transcription. We had earlier shown that ORF45, an immediate-early protein [24] and a virion component (tegument) protein of KSHV [25], [26], interacts with IRF-7 and suppresses its activation, thereby efficiently inhibiting the virus-mediated type I IFN induction [27]. Thus, ORF45 might be critical in defeating the host antiviral responses following primary KSHV infection. Tegument proteins even in closely related herpesviruses like HCMV [11], [12] and Epstein-Barr virus (EBV) [28] provide for a similar role. In this report we thus investigated the possible role of ORF45 in overcoming the innate host immune responses. We first show that KSHV does not induce an antiviral state following primary infection and also subsequently reveal the likely virion component responsible for this to be the tegument protein ORF45. We also show that IRF-7 critically contributes to the primary immune response elicited against incoming KSHV particles. Thus this study convincingly illustrates the biological significance of the ORF45-IRF-7 interaction following primary infection of cells with KSHV viral particles.

## Results

### KSHV does not induce an antiviral state during *de novo* infection

Viral infection and ongoing lytic replication are generally under host antiviral surveillance. These viral events induce specific antiviral immune responses by the host cell constituted by the synthesis of type I IFNs (IFN-α/β) that activates the transcription of a group of downstream ISGs that encode antiviral IFN effector proteins [15]. To assure successful infection and lytic replication, many viruses including related herpesviruses like HSV-1, HCMV, RhCMV and EBV have developed strategies to specifically evade these elicited host immune responses [11]–[14], [28]. Thus, we wanted to investigate if KSHV is also able to avoid such host antiviral responses following primary infection. To address this question, we adapted a plaque reduction assay in our study. A similar assay also has been used to demonstrate that herpes simplex virus type I (HSV-1) is able to disarm IFN antiviral responses [14]. KSHV virions collected five days following 12-*O*-tetradecanoylphorbol-13-acetate (TPA) induction of BCBL-1 cells (harboring KSHV in the latent phase), were concentrated and gradient purified. A portion of these virions were subjected to UV-irradiation. Human foreskin fibroblast (HFF) cells were infected with 5 genome copies per cell of intact or UV-inactivated KSHV and at 16 hours postinfection were superinfected with approximately 100 plaque forming units (Pfu) of vesicular stomatitis virus (VSV). VSV is a negative strand RNA virus, the growth of which is sensitive to the effects of the IFN pathways, hence has been commonly employed as a useful indicator to assess the antiviral status of a cell following herpesviral infection [8], [14]. VSV replication is indicated by formation of characteristic plaques which upon crystal violet staining allows for convenient visualization.

**Figure 1:**
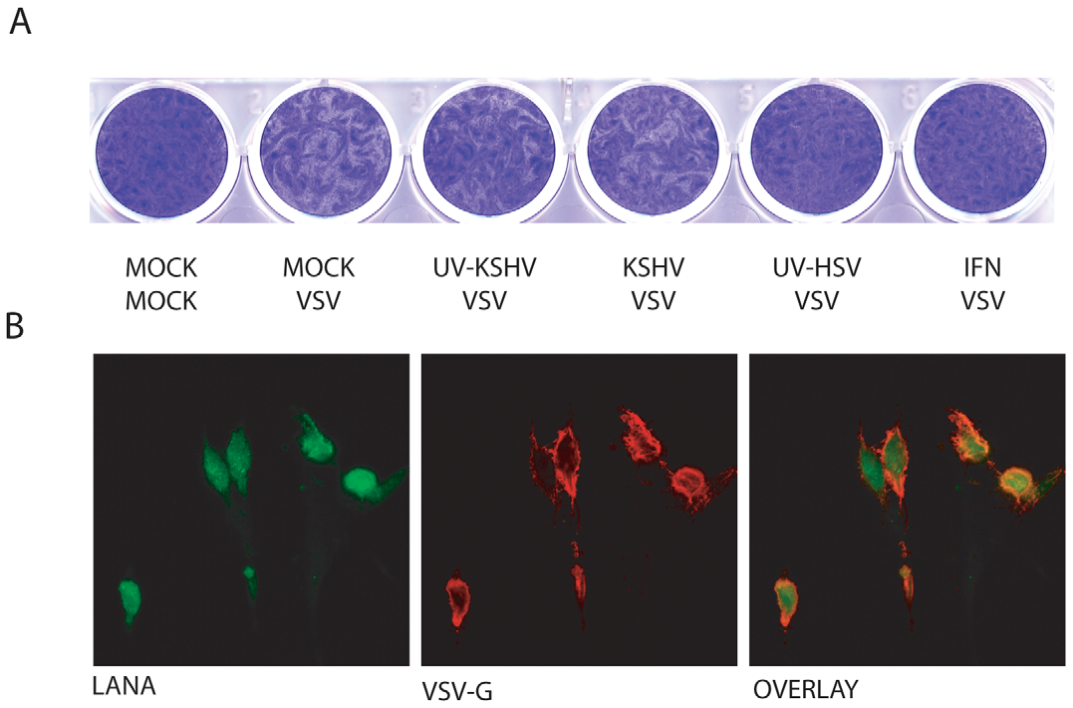
Evaluation of antiviral state subsequent to KSHV primary infection. (**A**) Human foreskin fibroblast (HFF) monolayers were mock-infected or infected with intact/UV-inactivated KSHV virions (5 viral genomes/cell) or UV-inactivated HSV-1 (multiplicity of infection [MOI] of 5) or treated with IFNα (1000 units/ml). After 16 h, 100 plaque forming units (Pfu) of vesicular stomatitis virus (VSV) was added to each well and then overlayed with 1% methylcellulose in DMEM. Cells were fixed and stained with crystal violet 24 h later for convenient visualization of VSV induced plaques. (**B**) Confocal immunoflorescence revealing dual infection of HFF cells with KSHV and VSV. HFF cells grown on coverslips were infected first with intact KSHV virions and subsequently superinfected with VSV. Cells were fixed, permeabilized and subjected to a double labeled immunoflorescence (IFA) with mouse monoclonal antibody against KSHV LANA (nuclear green florescence) and rabbit polyclonal antibody against VSV glycoprotein (cytoplasmic red florescennce). The right panel shows the merged image.

HFF cells that were infected with either UV-irradiated HSV-1 (MOI of 5) or IFNα (1000 units/ml) resisted VSV superinfection as shown by the near absence of VSV specific plaques (Fig. 1A). This was in agreement with earlier findings wherein HSV primary infection elicits immune responses which are disarmed only following immediate-early gene expression [14]. Interestingly in KSHV-infected cells, no apparent reduction of VSV plaques was observed regardless of UV treatment of KSHV (Fig. 1A). Even when HFF cells were infected with a higher MOI of KSHV (50 genomes per cell), similar results were obtained (data not shown). Merged/overlayed confocal immunoflorescence images revealed the presence of both KSHV latent nuclear antigen, LANA (green nuclear staining) and VSV glycoprotein G, VSV G (red cytoplasmic staining) in the same cell (Fig. 1B). These results were in corroboration to an earlier study [9] thereby suggesting that primary infection with KSHV dose not lead to an antiviral state and permits subsequent superinfection of KSHV-infected cells with VSV.

**Figure 2:**
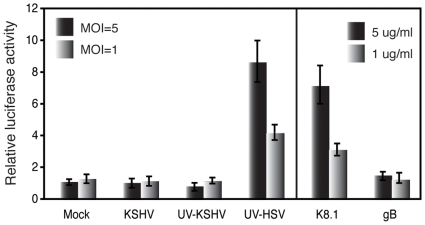
Induction of IFN signaling by KSHV soluble KSHV K8.1 glycoprotein but not by KSHV virions. Subconfluent 293T cells grown in 24-well plates were transfected with pGL-IFNA1 (a luciferase report vector driven by the IFNA1 promoter), an IRF-7 expressing plasmid (pCR3.1-IRF-7) cloned into a pCR3.1 vector and a pRL-TK reporter (*Renilla* luciferase as an internal control) plasmid. At 24 h post-transfection, cells were infected with the indicated viruses at the indicated MOI or treated with purified soluble KSHV K8.1 or gB glycoproteins at increasing concentrations of 1 and 5 µg/ml, respectively. Six hours following infection, cell lysates were prepared and activation of the IFNA1 promoter transcription was evaluated using a dual luciferase assay kit.

The ablation of an effective antiviral response might rest in lack of molecular signals to trigger host cellular responses. However, modulation of several host cellular signaling pathways following primary infection with KSHV [10] combined with the fact that type I IFN mediated antiviral immune responses are known to be generated following infection with other related herpesviruses [7], [8], [11]–[14], [28] argues against this notion. Thus an alternative possibility of KSHV triggering an initial antiviral response which is quickly and efficiently disarmed seems much more feasible. Hence we next went on to investigate if an effective type I IFN based immune response is generated in the first place subsequent to KSHV primary infection. In related herpesviruses like HSV-1 and HCMV, binding of the envelope glycoproteins to the host cell surface, induces the type I IFN signaling [7], [8]. To investigate occurrence of a similar such phenomenon in KSHV too, we used a sensitive luciferase-reporter system to determine whether KSHV major glycoproteins K8.1 or gB activates the type I IFN pathway. Human embryonic kidney (HEK) 293T cells co-transfected with an IFNA1 promoter reporter construct (pGL-IFNA1) and an IRF-7 expression vector (pCR3.1-IRF-7) were infected 24 h later at increasing inputs of either purified envelope glycoproteins (K8.1 or gB) or gradient purified intact or UV-inactivated KSHV. As expected, challenge of cells with the UV-irradiated HSV-1 (control) at increasing MOIs of 1 and 5 proportionately increased the IFNA1 promoter transcription 4-fold and 8-fold respectively (Fig. 2). Interestingly increasing inputs of the KSHV K8.1 protein, corresponding to 1 and 5 µg/ml, concomitantly increased the IFNA1 promoter activity 3 and 7-fold respectively, though purified gB had no such effect at both concentrations (Fig. 2). Both intact and UV-treated KSHV virions did not reveal any noticeable increases in the IFNA1 promoter being comparable to the mock infected cells (Fig. 2). These results demonstrated that the KSHV envelope glycoprotein K8.1 triggers host antiviral signaling which is quickly ablated by some virion component(s).

### KSHV tegument protein ORF45 is delivered into cells during *de novo* infection

**Figure 3:**
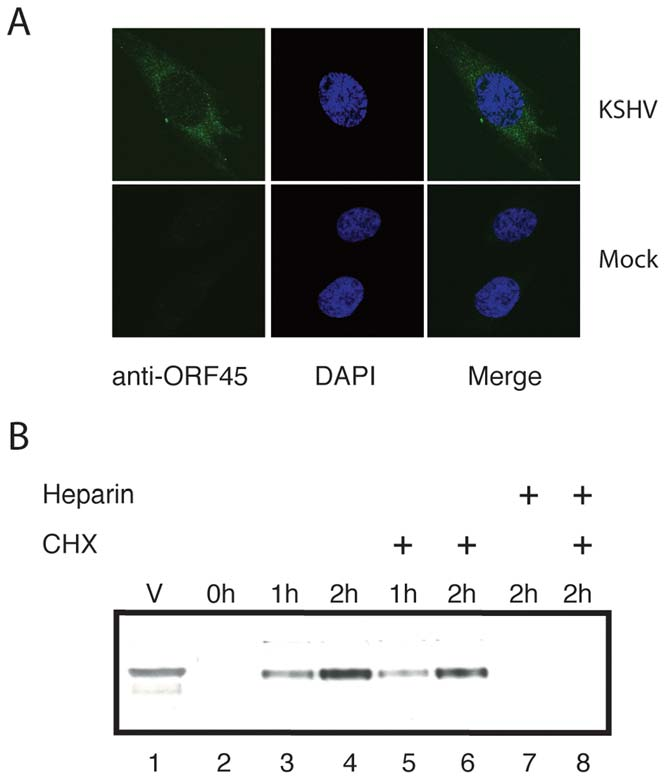
KSHV tegument protein ORF45 is delivered into cells during *de novo* viral infection. (**A**) HFF monolayers were mock infected or infected with gradient-purified KSHV virions. At one hour post-infection, these cells were subjected to an immunoflorescence (IFA) by staining with mouse monoclonal antibody (2D4A5) against ORF45 followed by subsequent treatment with Alexa-488 conjugated anti-mouse secondary antibody and counterstaining with DAPI. The DAPI panel shows the nucleus counterstained with DAPI. (**B**) 293T cells were infected with gradient-purified KSHV virions in the absence (lanes 3, 4) or the presence of cycloheximide (CHX) (lanes 5 and 6) or heparin (lanes 7 and 8). After 1 or 2 h post-infection, the unbound viruses were washed away with low pH buffer. Cell extracts were made and protein concentration was measured using a Bradford assay kit (Bio-Rad). About 50 µg of the protein extract was run on 4–12% Bis-Tris gels and expression levels of ORF45 were detected by a western blot using a specific mouse monoclonal anti-ORF45 antibody. The viral inoculum lysate (lane 1) and mock-infected cell lysate (lane 2) served as controls.

Given that a virion component likely disarms the initial host elicited immune response, we hypothesized that a highly probable virion protein to mimic this could be ORF45. This was based on two earlier observations: (i) ORF45 is a major component of the KSHV viral tegument [25], [26]; (ii) ORF45 characteristically inhibits the activation of IRF-7 [27], the master regulator essential for both the initial and the late phase induction of type I IFNs [20], [21]. As shown in Fig. 3A, ORF45 was readily detected by immunoflorescence as early as 1 h following infection of HFF cells. Similar findings were also reproducible following infection of both 293T and Vero cells (data not shown). Western blot analysis using infected cell lysates also revealed ORF45 in KSHV-infected cells as early as 1 hr post infection (Fig. 3B, lanes 3 and 4). A series of observations confirmed that the ORF45 detected is only the virion-associated form and not newly synthesized: (i) infection of cells with UV inactivated KSHV virions also revealed ORF45 as seen with intact viruses (data not shown); (ii) treatment of cells with cycloheximide (a well characterized protein biosynthesis inhibitor) did not abrogate detection of ORF45 (Fig. 3B, lanes 5 and 6). Furthermore, treatment of cells with heparin (shown to block KSHV entry into cells, [29], [30]) revealed the complete absence of ORF45 (Fig. 3B, lanes 7 and 8). These findings thus confirmed that ORF45 is delivered directly to *de novo* infected cells immediately following infection. This combined with the fact that ORF45 being a tegument component [25], [26] is virtually exposed to the cell environment thus makes it suitably poised to manipulate the host immune response.

### ORF45-null virus triggers an antiviral state in human fibroblast cells

Due to the several observations that favored a possible role of ORF45 in antagonism of the host antiviral responses, we went on to further investigate its potential role. Towards this by genetic recombineering methodology, we generated an ORF45-null KSHV recombinant virus (BAC-stop45) from a BAC-cloned wild-type KSHV genome (BAC36) [31] by introducing a premature stop codon into the ORF45 coding sequence in the KSHV genome [32], totally abolishing the functional ORF45 synthesis. The wild-type (BAC36 and revertant virus) and the mutant BAC DNAs were transfected into 293T cells and subjected to hygromycin selection that led to the generation of 293T monolayers stably transfected with either of the BAC DNAs. Viruses were reconstituted from these monolayer cultures subsequent to TPA induction [32]. The ORF45 null recombinant virus system thus provided us with utmost convenience and reliability to assess the hypothesized role of ORF45 in immune evasion.

**Figure 4:**
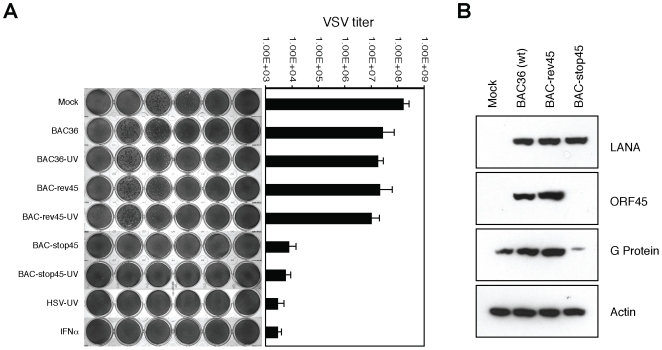
Induction of an antiviral state by the ORF45-null recombinant KSHV virus. (**A**) Human foreskin fibroblast (HFF) monolayers were mock infected (Mock) or infected with the KSHV recombinant viruses, including the wild-type BAC36, UV-irradiated BAC36 (BAC36-UV), BAC-rev45, UV-irradiated BAC-rev45 (BAC-rev45-UV), BAC-stop45, or UV-irradiated BAC-stop45 (BAC-stop45-UV) at 5 genomes/cell. HFF cells infected with UV-inactivated HSV-1 (HSV-UV) at an MOI of 5 or treated with IFNα (1000 units/ml) were also included as controls. The following day, the cells were superinfected with 100 plaque forming units (Pfu) of VSV. Media were collected 24 h later and VSV titers were determined by a standard plaque assay. Data are the average of the results from three experiments. Error bars represent standard deviation. (**B**) HFF monolayers mock infected or infected with BAC36 (wild-type, wt), BAC-rev45 or BAC-stop45 viruses were super infected as above with VSV. Twenty-four hours postinfection, cell lysates were collected and analyzed by a Western blot for expression levels of KSHV latent nuclear antigen (LANA), KSHV ORF45, VSV G glycoprotein and β-actin (loading control) employing specific antibodies.

To determine whether cells infected with ORF45-null virus show altered susceptibility to subsequent virus infection, VSV plaque formation was assessed in HFF cells infected with intact or UV-irradiated recombinant viruses, including wild type (BAC36), revertant (BAC-rev45) and ORF45-null mutant (BAC-stop45), followed by superinfection with VSV. The results showed that VSV superinfection produced relatively large numbers of plaques on mock-infected, BAC36 and BAC-rev45 (including UV-treated)-infected HFF cells, but few plaques on HFF monolayers earlier infected with BAC-stop45 or UV-treated BAC-stop45. No plaque was seen on cells challenged with UV-irradiated HSV-1 or treated with IFNα (data not shown). To quantitate the VSV superinfection in cells challenged with various agents, the experiment was repeated but with a slight modification. Instead of overlaying with methylcellulose, fresh medium was added to each well after superinfection with VSV and the VSV released into the medium was titrated by a plaque assay. As shown in Fig. 4A, VSV titers from cells infected with intact or UV-irradiated wild type KSHV (BAC36 and BAC-rev45) were appreciably high being only slightly lower than those obtained with the mock infected cells. However HFF cells treated with intact or UV-inactivated ORF45-null mutant virus (BAC-stop45) showed about a 3 log reduction in VSV titers.

Susceptibility of wild-type and ORF45 null virus infected HFF monolayers to VSV superinfection was also addressed by a Western analysis of the superinfected cells for VSV glycoprotein (G). As a reflection of the VSV plaque reduction assay, markedly reduced expression levels of VSV G glycoprotein was revealed in the cells infected initially with the ORF45-null recombinant virus compared to the BAC36 and BAC-rev45 infected cells (Fig. 4B). Collectively, these findings indicated the ability of an ORF45-deficient KSHV to trigger an antiviral state convincingly pointing out the role of ORF45 in subversion of host antiviral defenses.

### Antiviral state triggered by ORF45-null virus is associated with increases in type I IFN signaling

The antiviral state induced by infection with the ORF45-null recombinant virus, but not with wild type KSHV, thus suggested a rational association with concomitant increases in the host induced immune response pathways in host cells. Thus to investigate the occurrence of this phenomenon, induction levels of specific immune response genes (inclusive of IFNs, IFN receptors, IRFs and the IFN inducible proteins) obtained with both the wild-type and the ORF45 deficient viruses was conveniently and reliably measured by the employment of a commercial 96-well formatted SYBR-green based real-time PCR approach (Interferon [IFN] and Receptor PCR Assay from SABiosciences). Total RNAs collected 6 hours post infection of HFF monolayers with either the BAC36 or the BAC-stop45 viruses were reverse-transcribed to cDNA and subjected to a real-time PCR. This system by combining the advantage of real-time PCR with the ability of microarrays thus allowed for the simultaneous detection of 84 immune response genes. The expression level obtained for each gene with both the wild-type and the ORF45-null viruses was normalized to GAPDH. The fold change for a particular gene of interest was subsequently calculated by comparing normalized value of that gene between the two groups (ORF45-null recombinant vs. wild-type). Genes that revealed a fold change greater than 2 was considered significant as perceived usually (Supporting Information, Table S1).

The antiviral defense mechanisms elicited by the host against the invading virus are predominantly type I IFN mediated [5], [15]. Hence in addition to IFNα and its receptor (IFN AR1/AR2) genes, a majority of the other immune response genes showing a greater than 2-fold induction with the ORF45-null mutant virus compared to the wild-type virus were the IFNα inducible downstream ISGs (Table S1). Among these were the genes with proven antiviral activities like the myxovirus resistance A (MxA), 2′-5′ oligoadenylate synthetase 1 (OAS1), the RNA specific adenosine deaminase (ADAR), IFN stimulated gene (ISG) 56 (IFN-induced protein with tetratricopeptide repeat [IFIT1]), ISG 54 (IFIT2) and ISG60 [15], [33]–[37] as shown in Table S1.

**Figure 5:**
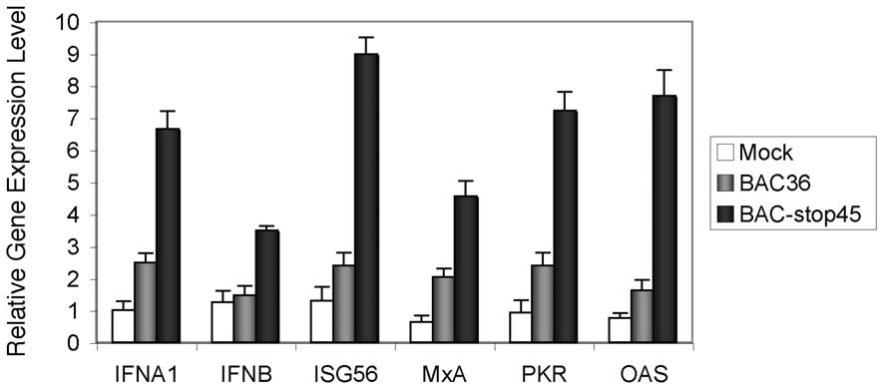
Evaluation of type I interferon (IFN) and downstream IFN stimulatory gene (ISG) transcription. HFF cells seeded in 6-well plates were infected with the KSHV wild-type (BAC36) or the ORF45-null recombinant (stop45) viruses. Six hours post-infection, cells were lysed with Trizol reagent and total RNAs isolated. Residual DNA contamination was eliminated by subsequent treatment with Turbo *DNase I* and the RNA was subsequently reverse transcribed to cDNA. The cDNA samples were then subjected to an absolute real-time PCR based quantification with specific primers for IFNA1, IFNB and selected downstream antiviral effector genes (ISG56, MxA, PKR and OAS). The amounts of mRNA were quantitated based on comparison with the standard templates of cloned cDNAs of known copy number following which the expression levels were normalized to GAPDH.

Some of the genes from the above group were subjected individually to absolute quantification by a real-time PCR employing specific primer sets (Fig. 5). These included the type I IFNs and a set of IFN induced downstream ISGs with potent antiviral effects (MxA, ISG56, OAS1 and protein kinase R [PKR]). PKR, a major antiviral effector, exerts its antiviral effects through inhibition of viral translation including in related herpesviruses like HSV [35]. At 6 hours post-infection, the levels of IFNβ (IFNB) remained nearly unchanged, while the levels of IFNα1 (IFNA1) and the other antiviral effectors like MxA, ISG 56, OAS and PKR were only marginally increased in cells infected with the wild type BAC36 virus compared to mock infected cells (Fig. 5). However the expression levels of IFNA1 and all the downstream antiviral effectors were found to be appreciably elevated in cells infected with the ORF45 null virus (with nearly a 4-fold increase seen with ISG56) (Fig. 5) compared to the BAC36 virus infected cells. The increased upregulation of ISG56 coincides well with the fact that it remains the most highly upregulated gene following IFNα induction [16]. The upregulation patterns of the type I IFNs and the downstream ISGs more or less mirrored each other in both the assays. Thus, it could be pointed out that the antiviral state induced by the ORF45-null mutant virus was associated in parallel with an induction of type I IFN mediated antiviral immune response genes.

A surprising observation is the low level activation of IFNβ following infection with the stop45 mutant virus (Table S1 and Fig. 5). It has been earlier shown in transgenic mouse models that in early phase immune response there is a predominance of IFNβ which subsequently gets replaced with IFNα [38] which could be mimicking our present observation which was observed only 6 hours post infection. The decreased potency of IFNβ in activating itself compared to its effect on IFNα combined with the finding that IFNα is potent in only activating itself following the initiation of the positive feedback loops have has been given as possible reasons [38].

### Ectopic expression of ORF45 in human fibroblast cells by lentivirus vector alleviates antiviral responses

**Figure 6:**
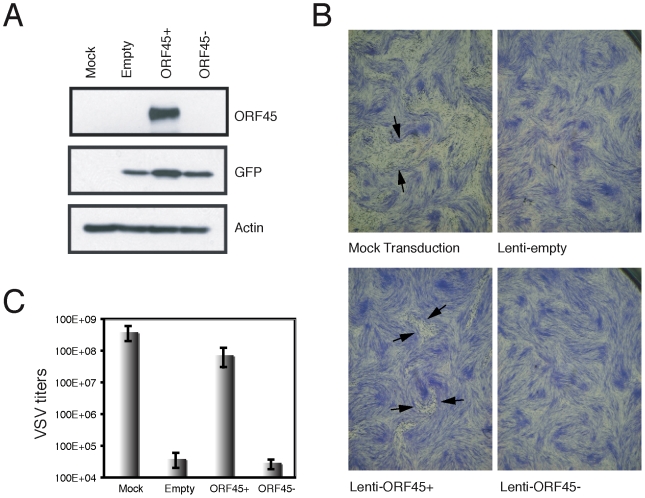
Ectopic expression of KSHV ORF45 in lentivirus transduced HFF cells permits VSV superinfection. HFF cells seeded in 24-well plates were mock-transduced or transduced with the lentiviruses expressing ORF45 (lenti-ORF45+) or not expressing ORF45 (lenti-empty and lenti-ORF45- [where the ORF45 sequence was inserted in the opposite direction]) as controls. (**A**) Cell lysates of lentivirus transduced cells were analyzed with Western blot with antibodies against ORF45 and GFP to ensure the success of transduction. The same blot was also probed with β-actin antibody for an equal loading control. (**B**) At 24 h post-tranduction, cells were superinfected with VSV, overlayed with 1% methylcellulose in media and stained with 0.1% crystal violet 24 h later for visulalization of plaques. Images were taken under 12x dissection microscopy. Some of the plaques in the mock transduced and the Lenti-ORF45+ transduced cell monolayers are indicated by arrows for easy visualization. (**C**) Quantitation of VSV super-infection. Parallel experiments were performed as above. After VSV superinfection, each well was supplemented with 1 ml fresh DMEM instead of being overlayed with methylcellulose. Media were collected 24 h later and VSV titers in the supernatant medium were determined by a standard plaque assay.

The loss-of-function assays clearly demonstrated the significant role of ORF45 in evasion of the host antiviral response during the initial stages of viral infection. Next, we examined whether ectopic expression of ORF45 in cells affects the susceptibilities of cells to VSV infection. HFF cells were transduced with the lentiviral vector expressing ORF45 (lenti-ORF45+) or with an empty lentiviral vector (lenti-empty) or with a vector in which ORF45 was inserted in the opposite orientation (lenti-ORF45-). Mock transduced HFFs were used as control. Transduction efficiencies were estimated to be more that 90% by examining green fluorescent protein (GFP) expression under fluorescent microscopy (data not shown) and successful lentivirus transduction was confirmed by Western blot analysis in which ORF45 expression was detected only in cells transduced with the lenti-ORF45+ (Fig. 6A). VSV plaques were clearly seen after superinfection in cells not transduced with lentivirus while cells transduced with the lentiviruses lenti-empty or lenti-ORF45- revealed no VSV plaque (Fig. 6B) indicating that the transduced lentiviral particles are capable of eliciting an immune response. In contrast, cells transduced with ORF45-expressing lentivirus (lenti-ORF45+) exhibited small but visible VSV plaques (Fig. 6B). Quantitation of VSV superinfection revealed a VSV titer of 4×10^8^ pfu/ml for non-transduced cells, whereas cells transduced with lenti-empty or lenti-ORF45^-^ yielded less than 4×10^4^ pfu/ml (Fig. 6C), suggesting that lentiviral infection interferes with subsequent VSV infection, consistent with the effect of HIV on VSV superinfection in monocytes [39]. The VSV titer from cells transduced with lenti-ORF45+ was 7.5×10^7^, slightly less than that of mock-transduced cells, but significantly higher than that of cells transduced with lentivirus not expressing ORF45 (Fig. 6C). Based on these data, we conclude that lentiviral infection of HFF cells induced an antiviral response, so that VSV titers were lower in lentiviruses-transduced cells than non-transduced cells (4×10^4^ vs. 4×10^8^); ectopic expression of ORF45 alleviates the antiviral response and restores the infectivity of VSV (7.5×10^7^ vs. 4×10^8^). Together, the loss-of-function and gain-of-function assays indicate that ORF45 is potent to antagonize the host antiviral response triggered by a viral infection.

### IRF-7 critically contributes to the innate immune response subsequent to primary KSHV infection

**Figure 7:**
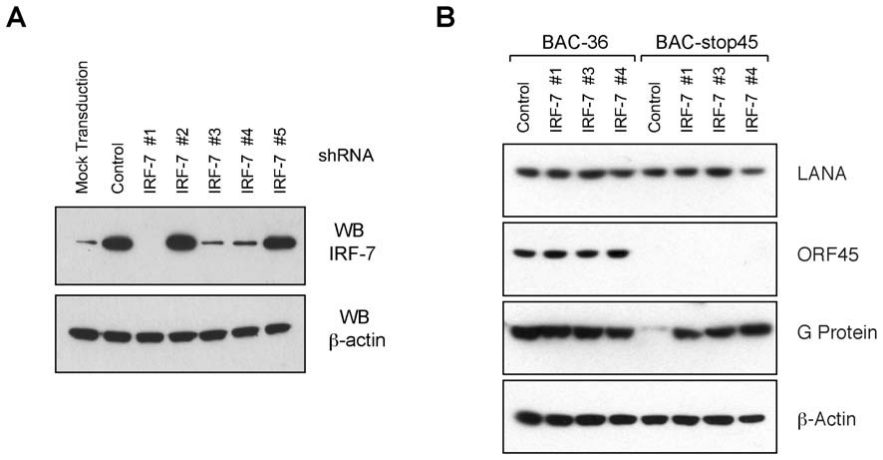
Interferon regulatory factor-7 (IRF-7) critically contributes to the immune response following primary KSHV infection. (**A**) Knockdown of IRF-7 expression by short-hairpin (shRNA)-mediated silencing. Five shRNA constructs (clone #s 1-5) each consisting of specific shRNA sequences in pLKO.1-puro plasmids against different target sites of IRF-7 mRNA along with a non-targeting “control” shRNA that activates the RNAi pathway without targeting any known human gene were transduced into HFF cells in the presence of polybrene. Transduced HFF cells were selected with puromycin (2 µg/ml) to allow for the generation of HFF monolayers expressing stable and long term downregulation of IRF-7. The efficiency of the different shRNA clones in knocking down expression of IRF-7 as compared to the “control” was tested by a Western blot (WB) performed on cell extracts with a rabbit polyclonal antibody against IRF-7 and subsequent probing with β-actin (equal loading control). Endogenous expression levels of IRF-7 in mock transfected HFF cells was also checked. (**B**) HFF monolayers expressing efficient down regulation of IRF-7 (clone #s 1, 3 and 4) along with the “control” transduced HFF cells were infected with the wild-type (BAC36) and the ORF45-null (BAC-stop45) recombinant viruses and subsequently super infected with VSV. Twenty four hours post VSV infection, cell lysates were collected and by a Western blot were analyzed for expression levels of KSHV latent nuclear antigen (LANA), KSHV ORF45, VSV G glycoprotein and β-actin (loading control) employing specific antibodies.

With ORF45 being shown to be the active KSHV virion component that effectively evades the host immune response combined with the earlier finding that it inactivates IRF-7, we finally wanted to investigate the role of IRF-7 in the induction of immune responses to the incoming KSHV and address the question if wild type KSHV indeed uses ORF45 to target IRF-7 in order to disarm elicited host antiviral responses. To this end, we attempted to knockdown the endogenous IRF-7 expression in HFF cells through a short-hairpin RNA (shRNA) approach. A Mission shRNA gene set against human IRF-7 gene was purchased from Sigma-Aldrich. The Mission shRNA system is a lentiviral vector-based RNA interference library against annotated human genes, which generates siRNAs in cells and mediates gene specific RNA interference for extended periods of time. The IRF-7 shRNA set consisted of 5 (clone #s 1-5) individual shRNA lentiviral vectors against different target sites of the IRF-7 mRNA sequence. HFF cells were individually transduced with these shRNAs and subjected to puromycin selection. The endogenous expression levels of IRF-7 in mock-transduced HFF cells were very low as shown by a Western analysis (Fig. 7A). The HFF cells that were transduced with a lentiviral “control”, (a shRNA sequence that activates the RNAi pathway without targeting any of the known human genes) resulted in increased levels of IRF-7 (Fig. 7A). This is consistent with previous studies that have shown that shRNA encoding vectors including shRNAs delivered by lentiviruses [40], [41] are capable of IFN activation that could generate a feedback loop resulting in increased IRF-7 levels. HFF monolayer cultures stably transduced with IRF-7 shRNA clone #1 gave almost a total knockdown of IRF-7, while cells transduced with clone #s 3 and 4 also gave appreciable reduction of IRF-7 (Fig. 7A). Thus these HFF monolayers giving noticeable IRF-7 knockdown (clone #s 1, 3, 4) along with the cells transduced with the “control” shRNA lentivirus were subsequently infected with either the BAC36 or the ORF45-null recombinant viruses followed by VSV superinfection as described above. The HFF cells that were transduced with IRF-7 shRNA clones 1, 3 and 4 and infected with ORF45-null mutant virus failed to induce any immune response as evidenced by the obvious susceptibility of these cells to superinfection with VSV. This was indicated by a Western blot revealing elevated expression levels of the VSV G glycoprotein in these cells (Fig. 7B). In contrast, the cells that transduced with the “control” shRNA and infected with the same virus (ORF45-null) were able to develop an antiviral state as manifested by the failure to be superinfected with VSV. The VSV G protein levels in IRF-7 shRNA (clones 1, 3 and 4) transduced and ORF45-null virus infected HFF cells were however found comparable to those in the cells transduced with the “control” shRNA transduced and infected with wild type KSHV (BAC36) (Fig. 7B). Additionally, the similar VSV susceptibilities were observed in BAC36 infected cells in the presence (with control shRNA) and absence (with IRF-7 shRNAs) of IRF-7 (Fig. 7B). Taken together, these data prove the vital role of IRF-7 in the development of an antiviral state that inhibits VSV superinfection and also indicate that ORF45 is very much effective in targeting this immune response by inactivating IRF-7 amounting to a total knockdown as mimicked with the specific shRNAs.

## Discussion

### Host antiviral responses and viral immune evasion

Primary infection of a cell with a herpesvirus is initiated with the attachment of the viral glycoproteins to the host cell surface. This is followed by the release of the viral components (tegumented capsids) into the cell environment for their transportation to the nucleus and the subsequent onset of viral replication. The host cell however immediately responds to the viral invasion by generation of specific antiviral immune responses [5], [6]. This predominantly involves the induction of type I IFNs (IFN-α/β) which trigger the transcription of a group of downstream IFN stimulated genes (ISGs) and production of effector proteins [5], [6], [15]. These effector proteins exert their functions through multiple pathways aiming to inhibit the viral transcription/translation also enhancing the degradation of the viral mRNA, ultimately helping the cell to eradicate the virus [15].

Such elicitation of immune responses against the invading virus, is seen in related herpesviruses like HSV and HCMV, wherein the binding of glycoproteins gD and gB respectively to the cell surface receptors induce type I IFN signaling followed by downstream multiple ISG synthesis [7], [8]. In KSHV too, infection of cultured fibroblast cells with soluble envelope glycoprotein K8.1 was found to induce a type I IFN response evidenced by increased transcription of both IFNβ and downstream ISGs with antiviral effects like 2′-5′ OAS and ISG 54 [9]. This effect was also substantiated by our present study that illustrated proportionate increases in the transcription of IFNA1 promoter following infection of HEK 293T cells with increasing inputs of KSHV K8.1 glycoprotein.

In spite of the type I IFN enhancing effect of KSHV gpK8.1, the virus was able to effectively disarm this response thus assuring the success of its infection. An earlier gene profiling study examining the up regulation of cellular genes following primary KSHV infection of cultured cells, indicated the clear absence of any increased transcription of both the type I IFN genes and its receptors [10]. Further infection of cells with UV treated KSHV virions revealed almost undetectable levels of IFNβ transcripts (inclusive of the downstream ISG54) [9] and failed to activate the IFNA1 promoter transcription activity (our present study). More importantly infection of cultured fibroblast cells with UV-treated KSHV virion particles [9] or with untreated/UV treated wild type KSHV clearly resulted in abrogation of the antiviral state illustrated by subsequent superinfection of cells with an indicator virus, VSV (our present study). Taken together, the preceding observations obviously indicate the role of a possible virion component in quickly and efficiently disarming the initial host elicited immune response. Interestingly in HCMV and RhCMV, virion components were found to counteract the initially generated host immune response [11]–[13].

### Role of ORF45 in antagonizing host antiviral responses

We had earlier shown that KSHV tegument protein ORF45 inhibits both phosphorylation and nuclear translocation of IRF-7 needed for its activation and for the substantial induction of type I IFNs [27]. In the present study, we employed genetic approaches, both loss-of-function and gain-of-function, to investigate the role of ORF45 in the immune evasion pathway. We found that KSHV is able to prevent activation of host antiviral defense mechanisms upon infection, as cells infected with wild type KSHV were permissive to superinfection by VSV. In contrast, infection of cells with ORF45-null viruses, both untreated and UV-irradiated, resulted in induction of an antiviral state hence markedly decreasing the susceptibility of the cells to superinfection with VSV. This correlated with a higher level of expression of IFNα, and many ISGs in the ORF45-null virus infected cells. Among the IFNα responsive downstream effector ISGs were the proteins predominantly associated with major antiviral activities like MxA, OAS, ADAR and IFIT1 (ISG56) [15], [33]-[37]. Significance needs to be attributed to this finding given that activated MxA effectively binds and traps incoming viral nucleocapsids targeting them towards degradation, thus eradicating incoming viral complexes even before start of viral gene transcription [36]. Taken together, our study results indicate the importance of down regulation of both type I IFN and its responsive downstream ISGs following primary KSHV infection.

In addition to the loss-of-function study, we also attempted to establish the role of ORF45 in evasion of immune responses through ectopic expression of ORF45. ORF45 expressing lentiviral particles upon transduction of fibroblast cells produced a characteristic ablation of an antiviral state evidenced by increased susceptibility of cells to subsequent VSV superinfection contrasting to the increased resistance offered to VSV by empty lentiviral particles or particles encoding for a non functional ORF45. Thus the extraneous expression of ORF45 by functioning as a gain of function assay, together with the loss-of-function assays employing ORF45-null mutant virus, demonstrated a crucial role of KSHV ORF45 in evading the host immune responses. It is worthwhile to note that this gain of function study revealed an additional function to block interferon responses. In KSHV infection, the ORF45 protein is present before cell gets significant amplification of the interferon response. However, with lentiviral infection, interferon responses along with IRF activation have initiated prior to ORF45 expression, but ORF45 is still able to block the interferon response. This notion has been proven by an shRNA-mediated IRF-7 knockdown study in HFF cells (Fig. 7) indicating that that ORF45 is able to disarm an antiviral response that has been already elicited by lentiviral infection.

### The master regulator of IFN-dependent immune responses is hit by a master viral inhibitor

KSHV uses an array of mechanisms to evade host antiviral responses including IFN signaling pathways [42], [43]. Besides ORF45, other immune evasion mechanisms of KSHV involving the type I IFN pathway have been described. The KSHV genome encodes four IRF homologues, designated vIRFs. Some of the vIRFs such as vIRF-1,vIRF-2 and vIRF-3 have been shown to inhibit cellular IRF pathways through dominant negative effects interfering with the activities of IRF-3 or IRF-7 [44]–[49]. In addition, KSHV also encodes for an IL-6 homolog, the vIL-6 shown to overcome IFN-mediated cell growth arrest [50]. Recently, the product of the KSHV ORF10 has also been shown to block the type I IFN signaling [51]. An immediate-early protein, RTA/ORF50 was also reported to suppress IRF-7 activation by targeting IRF-7 gene products for proteosome-mediated degradation [52]. The redundancy of counter-IFN activities encoded by KSHV emphasizes the importance of antagonism of the type I IFN signaling to the KSHV life cycle. Despite this redundancy, our loss-of-function and gain-of-function studies positively indicate that ORF45 is critical in the evasion of antiviral responses following primary infection. The profound role of ORF45 in immune evasion may rest in two unique features of ORF45.

First, ORF45 targets a fundamental regulator, which is of paramount importance of both innate and adaptive immunity. IRF-7 is a master regulator of type I IFN-dependent immune responses. Recent studies employing IRF-7 deficient (IRF-7^−/−^) mice demonstrated that type-1 IFN induction is severely impaired in the deficient mice and these mice are vulnerable to viral infection [20], [21]. Accordingly the low level of endogenous IRF-7 (in uninfected cells) subsequent to the viral infection gets activated by phosphorylation and translocates to the nucleus mediating the initial phase induction of type I IFN. This in turn further activates IRF-7 initiating a positive feedback loop for the substantially increased late phase induction of type I IFNs. Thus by vitally participating in both the initial and the late phases of IFN secretion, IRF-7 emerged as the only known IRF significantly modulating the host innate immunity [20], [21]. Hence it was not surprising that IRF-7 was also one of the genes significantly elevated following infection with the ORF45 null recombinant virus in our present study.

The importance of IRF-7 in host cell antiviral responses was again proved in our shRNA-mediated IRF-7 gene silencing experiment. Cells with the IRF-7 shRNA sequences were not able to develop antiviral responses upon infection with ORF45-null mutant virus as compared to control cells (with no IRF-7 knockdown) in which infection with ORF45-null virus conferred cell resistance to VSV superinfection. This finding suggested the following: (i) critical contribution of IRF-7 towards the innate immune response as shown earlier [20], [21] (ii) negligible contribution of other IRFs including IRF-3 towards induction of the type I IFN responses.

Given the role of endogenous IRF-7 it might well be suggested that KSHV ORF45 could directly interact with and inhibit the activation of the low level endogenously expressed IRF-7 hence preventing even the initial induction of type I IFNs and thus the subsequent initiation of the positive feedback loop for the increased late phase induction of IFNs. More recently IRF-7 was also found essential for the type I IFN induction mediated by the TLR9 subfamily of proteins [20], [21]. Viral invasion of a cell activates TLR9 that subsequently recruits the adaptor protein, MyD88. This adaptor protein through exclusive activation of IRF-7, results in type I IFN secretion, governing the induction of CD8^+^ T-cell responses contributing to adaptive immunity in DCs. It has been suggested that DCs could be infected with KSHV and could play vital roles in the transmission and pathogenesis of KSHV [22], [23], though further studies are underway. Thus it is tempting to speculate that ORF45 through its targeting of IRF-7 could play vital roles in evasion of both innate and adaptive immunity.

Second, unique among the KSHV encoded genes contributing to IFN mediated immune evasion as described earlier, ORF45 is the only known virion component abundantly present in the KSHV virions localized to the tegument [25], [26]. In the present study, we showed the presence of ORF45 in infected cells at very early time points (as early as 1 hour) following viral infection with both wild-type and UV treated KSHV virions. Thus it could be reasoned that ORF45 being a tegument component gets immediately delivered *de novo* to infected cells hence is quickly and potently able to exert its effect. This combined with its potential to inactivate IRF-7, obviously places this protein at a much higher pedestal compared to others in immune evasion immediately following primary viral infection. This not only allows KSHV to establish a fruitful infection but also lays the foundation for further progression of the viral life-cycle. Interestingly, periodic viral reactivation from latency also would come under host surveillance which again needs to be effectively evaded. ORF45 being an immediate-early protein [24] is produced very early following lytic reactivation, hence is again favorably poised to encounter this immune response too.

### Other implications from ORF45-mediated immune evasion

Evasion of host antiviral responses is not only important for viral propagation, but also linked to viral tumorigenesis. Several features of IFNs suggest their close inter relationship with tumor-suppressor pathways: (i) IFNs possess cell growth suppression activities [53]; (ii) type I IFNs serve as a bridge between innate and adaptive immune systems which have elaborate mechanisms to eliminate virus infected and cancer cells [54]; (iii) Some IRFs, such as IRF-3 and IRF-1, act as tumor suppressor genes [55]–[57]; (iv)type I IFNs can boost the response of p53 to stress signals. It has been shown that p53 gene induction by IFN α/β contributes to tumor suppression, linking IFN pathways directly to host tumor suppression pathways [58]. Thus, evasion of host immunity and tumorigenesis might be “two sides of the same coin” [42] and ORF45 might also contribute to KSHV-associated tumorigenesis by inhibiting IFN signaling.

KSHV-infected cells were also found permissive for superinfection by other viruses in our study, which may also have implications for the viral pathogenicity. HIV-1 is a known risk factor for KS and reciprocal interaction between HIV-1 and KSHV has been found in cells co-infected with these viruses. Such interaction may have impact on KS development and AIDS progression [59]–[61]. Co-infection of KSHV with other viruses such Epstein-Barr virus is also very common in PEL cells [62]. Overall, the strategy of antiviral immune evasion by ORF45 may thus be directly associated with KSHV pathogenicity.

## Materials and Methods

### Cells, viruses, plasmids and reagents

BCBL-1, a latent KSHV-infected primary effusion lymphoma cell line [63] was maintained in RPMI 1640 medium. Human cervical carcinoma derived cell line (HeLa) [27], human embryonic kidney (HEK) 293T [32] and human foreskin fibroblasts (HFF24441) cells [64] were maintained in Dulbecco's modified Eagle's medium (DMEM). All cultures were supplemented with 10% heat-inactivated fetal bovine serum (FBS) and antibiotics.

Sucrose gradient-purified HSV-1 KOS strain was kindly provided by Drs. Gary Cohen and Roselyn Eisenberg (University of Pennsylvania). A portion of these virions were also subjected to UV irradiation employing an UV Stratalinker 2400 instrument (Stratagene) for a period of time sufficient to reduce the viral titers by a factor of 10^5^. For production of KSHV virions, BCBL-1 cells were induced with TPA for 5 days. Post induction, the medium was collected and cleared by centrifugation to remove cells and cell debris. The supernatant was filtered through 0.45-µm filters. Virions were subsequently concentrated and gradient purified from the supernatant as per established protocols [25], [26]. A portion of KSHV virions were also irradiated by UV treatment as done for HSV. VSV (Indiana strain) was obtained from Dr. Ronald Harty (University of Pennsylvania). The virus was propagated and titers were determined by a standardized plaque reduction assay on Vero cells. Sendai viruses were bought from Charles River Laboratories (Wilmington, MA).

The BAC36/DH10B, containing the entire KSHV genome as a bacterial artificial chromosome (BAC) was obtained from Dr. S. J. Gao (University of Texas, San Antonio) [31]. Plasmids pGL-IFNA1, pCR3.1-IRF-7 and pCR3.1-ORF45 have been earlier described [27]. Purified recombinant K8.1 and gB proteins produced in a baculovirus expression system were kindly provided by Dr. Bala Chandran (University of Kansas Medical Center) [29], [65]. Human IFNα was a gift from Hoffmann-La Roche Inc. (Nutley, NJ). 12-0-tetradecanoyl-phorbol-13-acetate (TPA), sodium butyrate and polybrene (Hexadimethrine Bromide) were purchased from Sigma (St. Louis, MO). Hygromycin was purchased from Roche Inc. (Indianapolis, IN) and Turbo *DNase I* was obtained from Ambion (Austin, TX).

### Production of BAC36 wild-type and ORF45-null recombinant KSHV viruses

The detailed protocol for generation of recombinant KSHV with bacterial artificial chromosome (BAC) technology is described in Zhou et al [31]. To generate an ORF45-null mutant virus, we employed a two-step replacement procedure using the wild-type KSHV cloned into a BAC (BAC36) as the template [32]. In the first step, ORF45 coding sequence from the BAC36 genome was replaced with Kan/SacB double selection cassette by genetic recombineering. In the second step, a revertant (BAC-rev45) was generated by replacing the Kan/SacB cassettes with wild type ORF45 coding sequence. Similarly, an ORF45-null recombinant (BAC-stop45) virus was also generated by the introduction of a premature stop-codon (by replacing the 8th codon [TCG] in the ORF45 coding sequence to a stop codon, TAG) [32]. Wild-type BAC36, BAC-rev45 and BAC-stop45 DNAs were prepared using the Large Construct Kit (Qiagen). These freshly prepared BAC DNAs were transfected into 293T cells with the Qiagen Effectene transfection reagent and stable BAC-293T monolayers were generated through hygromycin selection as detailed earlier [32]. To produce recombinant viruses, monolayers were treated with 20 ng/ml of TPA and 0.3 mM sodium butyrate for induction of viral lytic replication. At 4–5 days post-induction, culture supernatants were collected and filtered through 0.45 µm filters. Virions were pelleted at 100,000 g for 1 h on a 25% sucrose cushion with a Beckman SW28 rotor. The pellets were dissolved in PBS to 1% of the original volume and the viral aliquots frozen at −80°C. Genomic copy number was subsequently estimated by a real-time PCR.

### Real-time PCR of viral genomic DNA

Concentrated viruses were first treated with Turbo *DNase I* at 37°C for 1 h to remove any contaminating DNA outside the viral particles. Viral DNA was liberated by subsequent digestion with lysis buffer (A) and proteinase K (supplied with the DNeasy tissue kit, Qiagen) and extracted with phenol-chloroform. Extracted DNA was precipitated with ice-cold ethanol, and the final DNA pellet was dissolved in Tris-EDTA buffer. Copy numbers of KSHV genomic DNA were estimated by a real-time DNA PCR with a Roche LightCycler using the LightCycler FastStart DNA Master^Plus^ SYBR green kit with primers directed to LANA [66]. Viral DNA copy numbers were calculated from external standards of known concentrations of BAC36 DNA. A serial dilution of a known amount of BAC36 DNA was used to construct a standard curve and copy numbers of KSHV genome (for downstream infection of cells) were determined by comparison to this standard curve.

### Plaque reduction assay

HFF2441 cells were seeded in 24-well plates to obtain a near confluent monolayer the next day. These monolayers were either mock-infected or infected with (i) intact/UV-irradiated KSHV virions (ii) intact/UV-irradiated KSHV recombinant viruses (BAC36 wild-type, BAC-rev45 and BAC ORF45 null [BAC-stop45]) at a multiplicity of infection (MOI) of 5 or 50 in serum-free DMEM for 1 h followed by replacement with DMEM containing 5% FBS. IFNα was added to mock-infected samples at 1000 units/ml. After 24 h, monolayers were super infected with approximately 100 plaque forming units (Pfu) of VSV, followed by replacement with DMEM containing 1% methylcellulose. Cells were fixed and stained with 0.1% crystal violet 24 h later for convenient visualization of the VSV induced plaques. In some cases, instead of overlaying with methylcellulose, fresh medium was added and cultures incubated for an additional 24 hours. Subsequent to this, the VSV titers in the supernatant medium were estimated by a standard plaque assay.

### Immunoflorescene assay (IFA)

For immunofluorescence analysis, HFF cells grown on coverslips were infected with KSHV followed with or without superinfection with VSV. Infected cells were fixed with 3% paraformaldehyde in PBS for 20 min and subsequently permeabilized with 0.1% Triton X-100 in PBS at 4°C for 10 min. Permeabilized cells were blocked with PBS (with 2% BSA) for 30 min at room temperature. Cells infected first with KSHV followed by VSV superinfection were subjected to a double-labeled IFA with mouse monoclonal anti-LANA (kindly provided by Bala Chandran) and rabbit anti VSV-G antibodies (Sigma). Alexa 488 conjugated goat anti-mouse IgG and Alexa 568 conjugated goat anti-rabbit IgG (Molecular Probes-Invitrogen Corp., Carlsbad, CA) were used as the respective secondary antibodies and cells were subsequently examined under a confocal microscope. On the other hand, cells infected only with KSHV were subjected to an IFA with a mouse monoclonal anti-ORF45 antibody followed by staining with Alexa 488 conjugated goat anti-mouse IgG and examined under a confocal microscope.

### Luciferase assay

Subconfluent 293T cells grown in 24-well plates were transfected with pGL-IFNA1 (a luciferase report vector driven by the IFNA1 promoter), an IRF-7 expressing plasmid (pCR3.1-IRF-7) cloned into a pCR3.1 vector and a pRL-TK reporter (*Renilla* luciferase as an internal control) plasmid by employing a Qiagen Effectene transfection kit. Twenty four hours subsequent to transfection, the cells were infected with the indicated viruses at specified MOI or with purified KSHV envelope glycoprotein K8.1 or gB at increasing inputs of 1 and 5 µg/ml respectively. Six hours post infection, cell lysates were collected and the luciferase assay was performed with the Promega's Dual-luciferase assay kit (Promega, Madison, WI).

### Western blot analyses

Monolayers of (i) 293T cells infected with gradient purified KSHV virions (ii) HFF cells infected with BAC cloned recombinant viruses (including the BAC36, BAC-rev45, BAC-stop45) followed by VSV superinfection at indicated time points were washed twice with ice-cold PBS and lysed with ice-cold lysis buffer (50 mM Tris-HCl [pH 7.4], 150 mM NaCl, 30 mM NaF, 5 mM EDTA, 10% glycerol, 40 mM α-glycerophosphate, 1 mM phenylmethylsulfonyl fluoride [PMSF], 1% Nonidet P-40, 1 mM sodium orthovanadate) supplemented with protease inhibitor cocktail (Roche). The cell lysates thus prepared were homogenized, clarified by high-speed centrifugation at 4°C and subsequently run on SDS-PAGE 8 to 12% Bis-Tris gels (Invitrogen) and transferred onto nitrocellulose membranes. These membranes were blocked in 5% dried milk in 1× phosphate-buffered saline plus 0.2% Tween 20 and subjected to a western blot using antibodies appropriate for the different proteins. Thus whole cell lysates prepared from (i) KSHV infected 293T cells were probed with mouse monoclonal anti-ORF45 antibody (ii) BAC cloned recombinant KSHV virus infected HFF cells were probed with mouse monoclonal anti-ORF45 [25], [26], rabbit polyclonal anti-LANA, rabbit polyclonal anti-VSV G (Sigma) along with mouse monoclonal anti-beta actin (Sigma) antibodies. Anti-rabbit or anti-mouse IgG antibodies conjugated to horseradish peroxidase (Amersham) were used as the respective secondary antibodies followed by an enhanced chemiluminescence system (ECL) for the final detection of the antibody-antigen complexes.

### Lentivirus-mediated expression of ORF45 in human fibroblast cells

Standard protocols were followed to generate HIV-based lentivirus expressing KSHV ORF45 [67]. The coding sequence of ORF45 was excised from pCR3.1-ORF45 and cloned into a lentivirus transfer vector. Two types of clones with both orientations were obtained. Clone 14, in which ORF45 was inserted with sense orientation, was used to generate lenti-ORF45+ that expresses ORF45 upon transduction. Clone 1, in which ORF45 was inserted with opposite orientation, was used to generate lenti-ORF45- that does not express ORF45 and hence was used as the negative control.

Lentivirus particles were generated in 293T cells using transient transfection with three plasmids: transfer vector containing ORF45 (either in the sense or in the opposite orientation), packaging plasmid pCMV8.2ΔR, envelope plasmid pVSVG [67]. Lentivirus released into the supernatant were harvested at 72 h post-transfection, centrifuged (500×g for 10 min at 4°C), filtered through a 0.45 µm filter to ensure removal of any non adherent cells and stored in aliquots at −70°C. Virus titers were subsequently determined by addition of serially dilutions of the lentiviral stocks to monolayers of HeLa cells grown in 96 well plate and positive infection determined by fluorescence microscopy.

HFF cells seeded in 24-well plates were transduced with the titered lentiviral stock in the presence of 4 µg/ml polybrene. Plates were centrifuged at 1,500×*g* for 90 min at room temperature to enhance the transduction process. Subsequently the transduced cells were washed with medium and cultured for an additional 24 h. After 24 h, monolayers were infected with approximately 100 plaque forming units (Pfu) of VSV and overlayed with 1% methylcellulose in media or in parallel supplemented with fresh DMEM. VSV viral titers were then obtained by standardized plaque assays as described earlier.

### Evaluation of transcription of type I IFNs and ISGs

HFF cells seeded in 6-well plates were either mock infected or infected with the KSHV wild-type (BAC36) or the ORF45-null recombinant (stop45) viruses at 50 genomes/cell. Six hours post infection, cells were lysed with Trizol reagent (Invitrogen) and total RNAs isolated according to the manufacturer's protocol. Residual DNA contamination was eliminated by subsequent treatment with Turbo *DNase I* (Ambion). cDNA was generated from total RNA with the SuperScript First-Strand Synthesis System (Invitrogen), priming with random hexamers. The cDNA thus obtained was analyzed by a commercial system employing a 96-well formatted SYBR-green based real-time PCR approach (Interferon [IFN] and Receptor PCR array [SABiosciences]) to compare the expression levels of type I IFNs and the type I IFN induced ISG levels in the cells infected with the BAC-stop45 viruses compared to those infected with BAC36. Herein the cDNA was mixed with a SYBR-green containing mix and aliquoted into wells of a 96-well plate (each well containing pre dispensed gene specific primer sets) and subjected to a real-time PCR by running on an ABI Prism7700 (Applied Biosystems) according to the manufacturer's instructions. This system combines the advantage of real-time PCR with the ability of microarrays hence allowing for the simultaneous detection of 84 immune response genes. The expression level obtained for each gene with both wild-type and the stop45 viruses was normalized to GAPDH. The fold change for a particular gene of interest was subsequently calculated by comparing normalized value of that gene between the 2 groups (ORF45 null recombinant vs. wild-type) as per the manufacturer's instructions. Genes that revealed a fold change greater than 2 were considered significant.

The cDNA samples were also subjected to absolute quantification by a real-time PCR to analyze the levels of type I IFNs and downstream antiviral effectors (ISG56, MxA, PKR and OAS) using primers specific for each gene as below:

- IFNA1+ (5′-TGATGAATGCGGACTCCATCT-3′)IFNA1- (GACAACCTCCCAGGCACAAG-3′)
- IFNB+ (5′-GCCAGAGGAATATGTCAGCTTT-3′)IFNB-(5′-GCAAAGTGATCCCCCAAATA-3′)
- ISG56+ (5′-AGGACTTCTAGCCTCGAGAACTT-3′)ISG56- (5′-GCTCCAGACTATCCTTGACCTG-3′)
- MxA+ (5′-ATCCTGGGATTTTGGGGCTT-3′)MxA- (5′-CCGCTTGTCGCTGGTGTCG-3′)
- PKR+ (5′-TCTACGCTTTGGGGCTAA-3′)PKR- (5′-GCCATCCCGTAGGTCTGT-3′)
- OAS+ (5′-AACTGCTTCCGACAATCAAC-3′)OAS- (5′-CCTCCTTCTCCCTCCAAAA-3′)
- GAPDH+ (5′-AGCCACATCGCTCAGACAC-3′,GAPDH- (5′-GCCCAATACGACCAAATCC-3')

Absolute quantification was performed with the Roche LightCycler using the FastStart DNA Master^Plus^ SYBR Green Kit. The amounts of mRNA were quantitated based on comparison with the standard templates of cloned cDNAs of known copy number following which the expression levels were normalized to GAPDH.

### Knockdown of IRF-7 expression using shRNA mediated silencing

Mission shRNA gene sets against the human IRF-7 were purchased from Sigma-Aldrich. This set consisted of five individual shRNA lentiviral vectors in pLKO.1-puro plasmids against different target sites of IRF-7 (with clone IDs NM_001572 -1213s1c1, -1156s1c1, -1573s1c1, -329s1ca, -1686s1c, for convenience sake referred to as clone #s 1-5). A “control” vector, SHC002 (a non-targeting shRNA that activates the RNAi pathway without targeting any known human gene) was also purchased (Sigma-Aldrich). Lentiviral stocks encoding the shRNA of interest were prepared by transient cotransfection of 293T cells with the shRNA encoding transfer vector and the two packaging vectors (pHR'8.2ΔR and pCMV-VSV-G) at a stipulated ratio of 4∶3∶1 respectively. Three days post-transfection, lentiviruses released into the medium were collected, harvested by centrifugation (500×g for 10 min at 4°C) and filtered through a 0.45 µm filter to ensure removal of cell debris and floating cells. Confluent monolayers of HFF cells were transduced with the shRNA encoding lentivirus stocks in the presence of polybrene (8 µg/ml). Transduced cells were selected with puromycin (2 µg/ml) to allow for the generation of HFF monolayers expressing stable and long term downregulation of IRF-7. Efficacies of the individual shRNAs in knocking down IRF-7 expression were evaluated by a western blot performed on the whole cell extracts probed with a rabbit polyclonal anti-IRF-7 antibody. HFF monolayers expressing efficient down regulation of IRF-7 were infected with the wild-type (BAC36) and the ORF45-null (BAC-stop45) recombinant viruses. Twenty-four hours post infection, cells were superinfected with VSV. Susceptibility of BAC36 and BAC-stop45 mutant infected HFF cells to VSV superinfection were analyzed for looking for expression levels of VSV-G protein by a Western blot on cell lysates probed with a rabbit polyclonal anti-VSV-G antibody.

## Supporting Information

Table S1 Comparison of Expression of Human Interferons and Their Responsive Genes between the Cells Infected with Wild-type (BAC36) and ORF45-null (BAC-stop45) Mutant KSHV Revealed by an RT^2^ Profiler PCR Array.
(0.15 MB DOC)
