# Supplementary material for: Antagonism of Host Antiviral Responses by Kaposi's Sarcoma-Associated Herpesvirus Tegument Protein ORF45
Source: PLoS One. 2010 May 11;5(5):e10573. doi: 10.1371/journal.pone.0010573 (PMC2868026; doi:10.1371/journal.pone.0010573)
Supplement: Table S1 — Comparison of Expression of Human Interferons and Their Responsive Genes between the Cells Infected with Wild-type (BAC36) and ORF45-null (BAC-stop45) Mutant KSHV Revealed by an RT2 Profiler PCR Array. (0.15 MB DOC) [file pone.0010573.s001.doc]

Table S1 - Comparison of Expression of Human Interferons and Their Responsive Genes between the Cells Infected with Wild-type (BAC36) and ORF45-null (BAC-stop45) Mutant KSHV Revealed by an RT^2^ Profiler PCR Array.

| Well | GeneBank | Symbol | Description | Gene Name | Fold Change Stop45 / BAC36 |
| --- | --- | --- | --- | --- | --- |
| G07 | NM_002462 | MX1 | Myxovirus (influenza virus) resistance 1, interferon-inducible protein p78 | IFI-78K/IFI78 | 5.30 |
| B10 | NM_001549 | IFIT3 | Interferon-induced protein with tetratricopeptide repeats 3 | CIG-49/GARG-49 | 3.95 |
| G01 | NM_006147 | IRF6 | Interferon regulatory factor 6 | LPS/OFC6 | 3.94 |
| D02 | NM_000416 | IFNGR1 | Interferon gamma receptor 1 | CD119/IFNGR | 3.58 |
| A01 | NM_001111 | ADAR | Adenosine deaminase, RNA-specific | ADAR1/DRADA | 3.55 |
| G02 | NM_001572 | IRF7 | Interferon regulatory factor 7 | IRF-7H/IRF7A | 3.54 |
| F02 | NM_000600 | IL6 | Interleukin 6 (interferon, beta 2) | BSF2/HGF | 3.46 |
| A08 | NM_001993 | F3 | Coagulation factor III (thromboplastin, tissue factor) | CD142/TF | 3.28 |
| G08 | NM_002534 | OAS1 | 2’,5’-oligoadenylate synthetase 1, 40/46kDa | IFI-4/OIAS | 3.15 |
| B07 | NM_001548 | IFIT1 (ISG56) | Interferon-induced protein with tetratricopeptide repeats 1 | G10P1/GARG-16 | 3.05 |
| B06 | NM_022168 | IFIH1 | Interferon induced with helicase C domain 1 | Hlcd/IDDM19 | 2.99 |
| F09 | NM_182972 | IRF2BP2 | Interferon regulatory factor 2 binding protein 2 | MGC72189 | 2.84 |
| F03 | NM_000565 | IL6R | Interleukin 6 receptor | CD126/IL-6R-1 | 2.52 |
| A12 | NM_005531 | IFI16 | Interferon, gamma-inducible protein 16 | IFNGIP1/PYHIN2 | 2.50 |
| C12 | NM_176891 | IFNE1 | Interferon epsilon 1 | IFNT1/PRO655 | 2.49 |
| C10 | NM_000874 | IFNAR2 | Interferon (alpha, beta and omega) receptor 2 | IFN-R/IFN-alpha-REC | 2.45 |
| C01 | NM_024013 | IFNA1 | Interferon, alpha 1 | IFL/IFN | 2.39 |
| B09 | NM_001547 | IFIT2 | Interferon-induced protein with tetratricopeptide repeats 2 | G10P2/GARG-39 | 2.18 |
| E12 | NM_000418 | IL4R | Interleukin 4 receptor | CD124/IL4RA | 2.15 |
| A11 | NM_002038 | IFI6 | Interferon, alpha-inducible protein 6 | 6-16/FAM14C | 2.10 |
| D06 | NM_001550 | IFRD1 | Interferon-related developmental regulator 1 | PC4/TIS7 | 2.08 |
| B05 | NM_006820 | IFI44L | Interferon-induced protein 44-like | C1orf29/GS3686 | 2.07 |
| D03 | NM_005534 | IFNGR2 | Interferon gamma receptor 2 (interferon gamma transducer 1) | AF-1/IFGR2 | 2.05 |
| C09 | NM_000629 | IFNAR1 | Interferon (alpha, beta and omega) receptor 1 | AVP/IFN-alpha-REC | 2.02 |
| B04 | NM_006417 | IFI44 | Interferon-induced protein 44 | MTAP44/p44 | 1.93 |
| G06 | NM_005373 | MPL | Myeloproliferative leukemia virus oncogene | C-MPL/CD110 | 1.79 |
| G05 | NM_002303 | LEPR | Leptin receptor | CD295/OBR | 1.79 |
| C07 | NM_021002 | IFNA6 | Interferon, alpha 6 | IFNA6 | 1.72 |
| F06 | NM_002198 | IRF1 | Interferon regulatory factor 1 | IRF-1/MAR | 1.67 |
| E11 | NM_002183 | IL3RA | Interleukin 3 receptor, alpha (low affinity) | CD123/IL3R | 1.67 |
| D09 | NM_000628 | IL10RB | Interleukin 10 receptor, beta | CDW210B/CRF2-4 | 1.64 |
| F04 | NM_002185 | IL7R | Interleukin 7 receptor | CD127/CDW127 | 1.63 |
| E03 | NM_021798 | IL21R | Interleukin 21 receptor | NILR | 1.62 |
| F08 | NM_015649 | IRF2BP1 | Interferon regulatory factor 2 binding protein 1 | DKFZP434M154 | 1.59 |
| F07 | NM_002199 | IRF2 | Interferon regulatory factor 2 | DKFZp686F0244/IRF-2 | 1.54 |
| G12 | NM_003319 | TTN | Titin | CMD1G/CMH9 | 1.54 |
| D12 | NM_001560 | IL13RA1 | Interleukin 13 receptor, alpha 1 | CD213A1/IL-13Ra | 1.52 |
| G09 | NM_176783 | PSME1 | Proteasome (prosome, macropain) activator subunit 1 (PA28 alpha) | IFI5111/PA28A | 1.49 |
| D07 | NM_006764 | IFRD2 | Interferon-related developmental regulator 2 | IFNRP/SKMc15 | 1.49 |
| A07 | NM_005755 | EBI3 | Epstein-Barr virus induced gene 3 | EBI3 | 1.46 |
| B11 | NM_003641 | IFITM1 | Interferon induced transmembrane protein 1 (9-27) | 9-27/CD225 | 1.38 |
| B02 | NM_006332 | IFI30 | Interferon, gamma-inducible protein 30 | GILT/IFI-30 | 1.37 |
| B03 | NM_005533 | IFI35 | Interferon-induced protein 35 | IFP35 | 1.16 |
| F12 | NM_002200 | IRF5 | Interferon regulatory factor 5 | IRF5 | 1.04 |
| G11 | NM_004509 | SP110 | SP110 nuclear body protein | IFI41/IFI75 | 1.03 |
| E01 | NM_000585 | IL15 | Interleukin 15 | IL-15 | 0.90 |
| A09 | NM_144717 | IL20RB | Interleukin 20 receptor beta | DIRS1/FNDC6 | 0.89 |
| F11 | NM_002460 | IRF4 | Interferon regulatory factor 4 | LSIRF/MUM1 | 0.86 |
| C11 | NM_002176 | IFNB1 | Interferon, beta 1, fibroblast | IFB/IFF | 0.85 |
| B12 | NM_006435 | IFITM2 | Interferon induced transmembrane protein 2 (1-8D) | 1-8D | 0.85 |
| D10 | NM_004512 | IL11RA | Interleukin 11 receptor, alpha | MGC2146 | 0.83 |
| A10 | NM_005101 | ISG15 | ISG15 ubiquitin-like modifier | G1P2/IFI15 | 0.77 |
| B01 | NM_005532 | IFI27 | Interferon, alpha-inducible protein 27 | FAM14D/ISG12 | 0.76 |
| F10 | NM_001571 | IRF3 | Interferon regulatory factor 3 | IRF-3 | 0.61 |
| C08 | NM_002170 | IFNA8 | Interferon, alpha 8 | IFNA8 | 0.48 |
| E10 | NM_139017 | IL31RA | Interleukin 31 receptor A | CRL/CRL3 | 0.05 |
| C03 | NM_000605 | IFNA2 | Interferon, alpha 2 | IFNA/INFA2 | 0.02 |
